# A personalized multi‐platform assessment of somatic mosaicism in the human frontal cortex

**DOI:** 10.1002/alz70855_103962

**Published:** 2025-12-23

**Authors:** Weichen Zhou, Camille Mumm, Yanming Gan, Jessica A Switzenberg, Jinhao Wang, Paulo De Oliveira, Kunal Kathuria, Steven J Losh, Torrin L McDonald, Brandt Bessell, Kinsey Van Deynze, Michael J McConnell, Alan P Boyle, Ryan E Mills

**Affiliations:** ^1^ University of Michigan Medical School, Ann Arbor, MI, USA; ^2^ Lieber Institute for Brain Development, Baltimore, MD, USA

## Abstract

**Background:**

Somatic mutations in individual cells lead to genomic mosaicism, contributing to the intricate regulatory landscape of genetic disorders and cancers. To evaluate and refine the detection of somatic mosaicism across different technologies with personalized donor‐specific assembly (DSA), we obtained tissue from the dorsolateral prefrontal cortex (DLPFC) of a post‐mortem neurotypical 31‐year‐old individual.

**Method:**

We sequenced bulk DLPFC tissue using Oxford Nanopore Technologies (∼60X), NovaSeq (∼30X), and linked‐read sequencing (∼28X). Additionally, we applied Cas9 capture methodology coupled with long‐read sequencing (TEnCATS), targeting active transposable elements. We also isolated and amplified DNA from flow‐sorted single DLPFC neurons using MALBAC, sequencing 115 of these MALBAC libraries on Nanopore and 94 on NovaSeq.

**Result:**

We constructed a haplotype‐resolved assembly with a total length of 5.77 Gb and a phase block length of 2.67 Mb (N50) to facilitate cross‐platform analysis of somatic genetic variations. We observed an increase in the phasing rate from 11.6% to 38.0% between short‐read and long‐read technologies. By generating a catalog of phased germline SNVs, CNVs, and TEs from the assembled genome, we applied standard approaches to recall these variants across sequencing technologies. We achieved aggregated recall rates from 97.3% to 99.4% based on long‐read bulk tissue data, setting an upper bound for detection limits.

Moreover, utilizing haplotype‐based analysis from DSA, we achieved a remarkable reduction in false positive somatic calls in bulk tissue, ranging from 14.9% to 72.4%. We developed pipelines leveraging DSA information to enhance somatic large genetic variant calling in long‐read single cells. By examining somatic variation using long‐reads in 115 individual neurons, we identified 468 candidate somatic heterozygous large deletions (1.5Mb ‐ 20Mb), 137 of which intersected with short‐read single‐cell data. Additionally, we identified 61 putative somatic TEs (60 *Alu*s, one LINE‐1) in the single‐cell data.

**Conclusion:**

Collectively, our analysis spans personalized assembly to single‐cell somatic variant calling, providing a comprehensive *ab initio ad finem* approach and resource in real human tissue.

